# Associations of household environmental tobacco smoke exposure with respiratory symptoms and utilisation of medical services in healthy young children in Hong Kong

**DOI:** 10.18332/tid/114461

**Published:** 2020-01-10

**Authors:** Siyu Dai, Kate C. Chan

**Affiliations:** 1 Department of Paediatrics, Faculty of Medicine, The Chinese University of Hong Kong, Hong Kong

**Keywords:** children, environmental tobacco smoke, medical care utilisation, respiratory tract disease

## Abstract

**INTRODUCTION:**

Young children are especially vulnerable to environmental tobacco smoke (ETS) exposure. This study was carried out to determine whether household ETS exposure was associated with respiratory symptoms and medical service utilisation among Hong Kong healthy children in their first eighteen months of age.

**METHODS:**

A secondary analysis was done on the data obtained from our previous cross-sectional territory-wide pneumococcal carriage surveillance study in Hong Kong in 2013–2014. All measures were reported by caregivers. Univariable and multivariable analyses were performed to examine the associations between ETS exposure and outcome measures. Covariates included children’s sex, age, body mass index z score, history of breastfeeding, gestational age at birth, birthweight, maternal age, living region, overcrowding of living area, household income, child care attendance, and presence of siblings. Additional adjustment for season and households’ respiratory symptoms were made to ascertain the association between ETS and children’s respiratory symptoms.

**RESULTS:**

The analysis included 1541 children (mean age: 11.2 ± 6.4 months; males: 50.7%). Current household ETS exposure (AOR=1.30; 95% CI: 1.00– 1.66; p=0.050) and postnatal maternal smoking (AOR=2.21; 95% CI: 1.06–4.64; p=0.035) were independently and significantly associated with all-cause doctor consultation in the past 3 months. Children living with more than one household smoker were more likely to have all-cause doctor consultation compared with the non-exposed children (AOR=1.70; 95% CI: 1.04–2.77, p=0.028). Postnatal maternal smoking was associated with all-cause hospitalisation in the past 3 months (AOR=2.48; 95% CI: 1.05–5.86; p=0.039). Children living in a household, where the daily consumption by household smokers was more than 20 cigarettes, were more likely to have respiratory symptoms compared with non-exposed children (AOR=1.99; 95% CI: 1.12–3.52; p=0.016).

**CONCLUSIONS:**

Household ETS exposure in young children was associated with respiratory symptoms and all-cause outpatient or inpatient medical service utilisation. The associations were potentially dose-dependent.

## INTRODUCTION

Environmental tobacco smoke (ETS) exposure in children is associated with various disease conditions, especially respiratory tract diseases like asthma and bronchiolitis^1^. A comprehensive systematic review concluded that the disease burden related to ETS exposure accounted for about 1% of worldwide mortality in 2004, with 28% of the affected population being children^2^.

Globally, the reported associations between children’s ETS exposure and all-cause medical service utilisation have been controversial^3-5^, while the positive associations were more consistently found in specific disease conditions such as asthma exacerbation^6^ and respiratory tract infections^7,8^. In Hong Kong, Lam et al.^9^ found *in utero* ETS exposure for non-smoking mothers was positively associated with more doctor consultations and hospitalisations in infancy, while postnatal exposure to ETS at home was associated with hospitalisations for any illness, but not observed for outpatient visits^9^. The impact of maternal smoking was not specifically examined in this previous study, while maternal smoking has a more significant impact on children’s health than the smoking of other household members^10,11^. Leung et al.^12^ found hospital admission was more prevalent among infants exposed to secondhand smoke if it was accompanied by poor smoking hygiene (<3 m away), however the results for smoking at least 3 m away from the infant was not statistically significant^12^. Moreover, they demonstrated that hospital admission of non-specified infectious disease was more likely among children exposed to ETS up to 8 years of age, if it was accompanied by poor smoking hygiene, and the association was the strongest in the first 6 months of life^13^. However, this study only investigated the association of ETS exposure with hospitalisation, which might have underestimated the impact of ETS on the utilisation of other medical services such as outpatient consultations. Furthermore, the dose-response relationships between exposure and health outcomes were less examined in previous studies^9,14^. The current evidence on the associations between ETS exposure and health problems in children is also limited by inadequate control for the potential effect of maternal smoking during pregnancy. Thus, it is worthwhile to further study the influence of ETS exposure among different groups of children, with or without a smoking mother.

Exposure to passive smoke significantly increases children’s risks of lower respiratory tract infection (LRTI), asthma, and reduced lung function^15-17^. LRTI in children aged <5 years was reported to be the largest global disease burden of ETS^2^. LRTI is also a leading infectious cause of mortality in children in this age group, worldwide^18^. Concerning the associations between ETS exposure and respiratory symptoms in children, wheezing was robustly linked to exposure, while controversial results were reported in studies about non-specific respiratory symptoms^19^. Some studies found ETS exposed children were more likely to have respiratory symptoms^20,21^, while others did not^22^. Parental respiratory symptoms were one of the important confounding factors that was not adequately addressed in previous studies. Furthermore, influence of season on children’s respiratory symptoms should be taken into consideration. Relationship between ETS exposure and medication consumption is rarely studied. Several recent European studies showed that ETS is associated with a higher antibiotic use rate in young children^23,24^, however the availability of such data in Hong Kong was limited.

With this study, we aimed to examine the associations between ETS exposure and utilisation of healthcare service, respiratory symptoms, and antibiotic use in young children in Hong Kong. We hypothesized that ETS exposed children had a higher risk of needing medical services, having respiratory symptoms, and taking antibiotics, compared with non-exposed children.

## METHODS

### Study population

This secondary analysis was performed on data collected from our 2013–2014 territory-wide cross-sectional pneumococcal carriage surveillance study, in which 1541 young children (mean age: 11.2 months; males: 50.7%) were recruited. Details of the surveillance study have been published^25^. Convenience samples were drawn. Children aged 2, 12 and 18 months were approached and recruited when they attended the immunization and health surveillance service provided by the Maternal and Child Health Centre across four main regions of Hong Kong. Screening for suitable subjects was done at the time of recruitment. Eligible children were invited to join the study with a response rate of 63.3%. Exclusion criteria were: 1) extreme low birth weight and very low birth weight infants; 2) subjects with cerebral palsy; 3) syndromes and neurological disorders affecting swallowing; 4) ear, nose and throat disorders; 5) confirmed or suspected immunodeficiency (congenital or acquired); 6) subjects taking immunosuppressive therapy; and 7) incomplete vaccination or vaccination record. This retrospective study was approved by the Joint Chinese University of Hong Kong–New Territories East Cluster Clinical Research Ethics Committee (CRE-2018.452).

### Data collection

Caregivers of the young children were asked to complete a questionnaire under supervision by a trained research nurse. The missing data rates were very low in the current study (less than 5% for all items). Data on a range of variables for the children were recorded including demographic information, household tobacco smoke exposure, medical service utilisation, respiratory symptoms, and antibiotic use.

### Exposure variables and disease outcome variables

Exposure binary variables were current household ETS exposure, prenatal maternal smoking exposure, and postnatal maternal smoking exposure. The caregiver was asked whether there were any smokers living with the child, whether mother smoked during pregnancy, and whether the mother was an active smoker (yes/no responses). Exposure level was examined using ordinal categorical variables including number of household smokers at home (0, 1, >1), and daily cigarette consumption by all household smokers (0, 1–20, >20). Disease outcomes were children’s medical service utilisation, presence of recent respiratory symptoms and antibiotic use. As for medical service utilisation, the caregiver was asked whether the child had a doctor consultation in the past 3 months (yes/no), with the major diagnosis for the visit recorded. Hospitalisations were recorded in a similar way. The caregiver was asked whether the child had respiratory symptoms in the past 1 month, and the specific symptoms were reported by the caregiver, which included fever, cough, sneezing, shortness of breath, sore throat, wheezing, middle-ear infection, and others. Antibiotic use in the past 3 months of the child was also recorded (yes/no). Our measures of ETS exposure and health outcomes were similar to previous studies^9,12,13^.

### Statistical method

Descriptive analysis was performed for children’s sociodemographic and clinical characteristics, medical service utilisation, and respiratory symptoms. Crude associations between the exposure variables and disease outcomes were analysed using chi-squared tests. As for the adjusted associations, all multivariable analyses had been adjusted for potential confounding factors: children’s sex, age, body mass index (BMI) z score, history of breastfeeding (yes/no), gestational age at birth, birthweight, maternal age, living region, overcrowding of living area (corresponding to a living space of <5.5 m^2^/person, in accordance with the guideline of the Hong Kong Housing Authority), low household income (household monthly income ≤HK$20000), childcare attendance (attendance of day care centre, nursery, or playgroup), and presence of siblings (yes/no). For respiratory symptoms, additional adjustment for household members’ respiratory symptoms and season were made. Multivariable logistic regression was used to evaluate the associations. The missing data rates were less than 5% for all items. The missing data were excluded in the analysis if there were any. As the number of missing data was very low, the effect on the results was basically negligible. A p-value of <0.05 was considered to be statistically significant, with p-values corrected (adjusted) for multiple tests (Bonferroni’s correction), where appropriate. All statistical analyses were performed by the SPSS package.

## RESULTS

### Demographic and clinical characteristics of the participants

The baseline characteristics, ETS exposure, medical service utilisation, respiratory symptoms and antibiotic use of the participants are shown in Table 1. More than half (61.6%) of the cohort had an all-cause doctor consultation in the past 3 months, and most of them were in private sectors. Six per cent of the children had hospitalisation in the past 3 months and the hospitalisation rate for respiratory tract infection (RTI) was 1.7%. Of the children, 19.4% took antibiotics in the past 3 months. One-third reported to have respiratory symptoms in the past 1 month.

**Table 1:** Demographic and clinical characteristics of the study participants, Hong Kong, 2013–2014 (N=1541)

*Characteristics*		All infants n (%)*
**Mean age** (months)		11.2 ± 6.4
**Male sex**		782 (50.7)
**Birth history**	Gestational age at birth (weeks)	38.6 ± 1.4
	Vaginal delivery	1030 (66.8)
	Birth weight (kg)	3.1 ± 0.4
**BMI z score**		0.2 ± 1.3
**Maternal age** (years)		32.7 ± 4.6
**History of breastfeeding**		1220 (79.2)
**Child care attendance**		197 (12.8)
**Presence of household smokers**	Any current household smoker	485 (31.5)
	Postnatal maternal smoking	53 (3.4)
	Prenatal maternal smoking	25 (1.6)
**Number of current household smokers**	0	1056 (68.5)
	1	377 (24.5)
	>1	108 (7.0)
	0	1056 (68.5)
**Cigarette consumption per day by household smokers**	1–20	430 (27.9)
	>20	55 (3.6)
**Medical service utilisation**	Any doctor consultation in the past 3 months	950 (61.6)
**Service type**	Consultation in private sector	769 (49.9)
	Consultation in public sector	93 (6.0)
	Consultation in both private and public sector	88 (6.0)
	Hospitalisation in the past 3 months	93 (6.0)
	Hospitalisation for RTI in the past 3 months	26 (1.7)
**Antibiotic use**	In the past 3 months	299 (19.4)
**Presence of respiratory symptoms**	In the past 1 month	515 (33.4)
**Presence of respiratory symptoms of household members**	In the past 1 month	5 (52.2)

* Values are number (%), unless specified as mean (±SD). RTI: Respiratory tract infection.

### ETS exposure and medical service utilisation

Need of doctor consultation was significantly higher among ETS exposed children, with an ascending trend for increasing exposure levels (Table 2). Children with current household ETS exposure (67.0 vs 59.2%; p=0.0030) and postnatal maternal smoking (81.1 vs 61.0%; p=0.0020) had higher rates of all-cause doctor consultation in the past 3 months compared with the non-exposed. Need of medical consultation was significantly larger as the exposure levels increased for both the number of household smokers and their total daily cigarette consumption. After adjustment of the p-values using Bonferroni’s correction, significance of associations remained (Table 3). Current household ETS exposure (AOR=1.30; 95% CI: 1.00–1.66; p=0.050) and postnatal maternal smoking (AOR=2.21; 95% CI: 1.06–4.64, p=0.035) were independently and significantly associated with all-cause doctor consultation in the past 3 months. Children living with more than 1 household smoker (AOR=1.70; 95% CI: 1.04–2.77; p=0.028) were shown to have higher risk of having all-cause doctor consultation compared with the non-exposed, however the association was not significant for children living with 1 household smoker. Postnatal maternal smoking was associated with all-cause hospitalisation in the past 3 months (AOR=2.48; 95% CI: 1.05–5.86; p=0.039).

**Table 2:** Univariable analyses: Rates of medical care utilisation associated with the ETS measures of the study participants, Hong Kong, 2013–2014 (N=1541)

*ETS exposure*		*Any doctor consultation in the past 3 months*			*Any hospitalisation in the past 3 months*		
		*n*	*%^a^*	*p*	*n*	*%*	*p*
Presence of household smokers	No	625	59.2		56	5.3	
	Yes	325	67.0	0.0030	37	7.6	0.084
Postnatal maternal smoking	No	907	61.0		86	5.8	
	Yes	43	81.1	0.0020	7	13.2	0.037
Prenatal maternal smoking	No	930	61.3		90	5.9	
	Yes	20	80.0	0.063	3	12.0	0.19
Number of household smokers	0	622	59.1		55	5.2	
	1	250	65.6	0.026	28	7.4	0.13
	>1	78	72.2	0.0090	10	9.3	0.093
Cigarettes per day by household smokers	0	623	59.2		56	5.3	
	1–20	283	65.8	0.017	33	7.7	0.083
	>20	40	75.5	0.021	4	7.5	0.49

a Rate of medical service utilisation in each ETS exposure variable condition.

**Table 3:** Multivariable analyses: Associations between ETS exposure and medical service utilisation of the study participants, Hong Kong, 2013–2014 (N=1541)

*ETS exposure*		*Any doctor consultation in the past 3 months*		*Any hospitalisation in the past 3 months*	
		*AOR (95% CI)*	*p*	*AOR (95% CI)*	*p*
Presence of household smokers		1.30 (1.00–1.66)	0.050	1.42 (0.90–2.25)	0.13
Postnatal maternal smoking		2.21 (1.06–4.64)	0.035	2.48 (1.05–5.86)	0.039
Prenatal maternal smoking		2.30 (0.80–6.57)	0.12	2.17 (0.61–7.67)	0.23
Number of household smokers	0	1		1	
	1	1.21 (0.93–1.59)	0.16	1.41 (0.86–2.30)	0.18
	>1	1.70 (1.04–2.77)	0.028	1.76 (0.83–3.74)	0.14
Cigarettes per day by household smokers	0	1		1	
	1–20	1.24 (0.96–1.61)	0.10	1.44 (0.90–2.31)	0.13
	>20	1.84 (0.92–3.68)	0.087	1.42 (0.47–4.25)	0.53

AOR: adjusted odds ratio; adjusted for children’s sex, age, body mass index (BMI) z score, history of breastfeeding, gestational age at birth, birthweight, maternal age, living region, overcrowding of living area, household income, child care attendance, and presence of siblings.

### Associations between ETS exposure and respiratory symptoms

Young children with household smokers consuming more than 20 cigarettes/day had significantly higher rate of having respiratory symptoms in the past 1 month compared with the non-exposed (47.2 vs 32.6%; p=0.030). The difference remained statistically significant after adjustment (Table 4).

**Table 4:** Associations between ETS exposure and presence of respiratory symptoms of the study participants, Hong Kong, 2013–2014 (N=1541)

*ETS exposure*		*Presence of respiratory symptoms in the past 1 month Univariable*			*Presence of respiratory symptoms in the past 1 month Multivariable*	
		*n*	*%^a^*	*p*	*AOR (95% CI)*	*p*
Presence of household smokers	No	342	32.4		1	
	Yes	173	35.7	0.22	2.95 (0.64–13.51)	0.16
Postnatal maternal smoking	No	499	33.5		1	
	Yes	16	30.2	0.66	0.68 (0.36–1.29)	0.23
Prenatal maternal smoking	No	507	33.4		1	
	Yes	8	32.0	1.0	0.95 (0.38–2.38)	0.92
Number of household smokers	0	343	32.6		1	
	1	132	34.6	0.47	1.07 (0.81–1.41)	0.62
	>1	40	37.0	0.35	1.15 (0.72–1.82)	0.56
Cigarettes per day by household smokers	0	343	32.6		1	
	1–20	144	33.5	0.73	1.03 (0.79–1.34)	0.85
	>20	25	47.2	0.030	1.99 (1.12–3.52)	0.016

a Rate of symptom development in each exposure variable. AOR: adjusted odds ratio; adjusted for children’s sex, age, body mass index (BMI) z score, history of breastfeeding, gestational age at birth, birthweight, maternal age, living region, overcrowding of living area, household income, child care attendance, presence of siblings, household members’ respiratory symptom, and season.

### Associations between ETS exposure and antibiotic use

No statistically significant associations between ETS exposure and antibiotic use were found in the univariable and multivariable analyses (Table 5).

**Table 5:** Associations between ETS exposure and antibiotic use of the study participants in Hong Kong, 2013– 2014 (N=1541)

*ETS exposure*		*Antibiotic use in the past 3 months Univariable*			*Antibiotic use in the past 3 months Multivariable*	
		*n*	*%^a^*	*p*	*AOR (95% CI)*	*p*
Presence of household smokers	No	192	18.2		1	
	Yes	96	19.8	0.78	0.90 (0.66–1.23)	0.50
Postnatal maternal smoking	No	265	17.8		1	
	Yes	15	28.3	0.11	1.26 (0.57–2.75)	0.57
Prenatal maternal smoking	No	271	17.9		1	
	Yes	7	28.0	0.31	1.61 (0.56–4.60)	0.38
Number of household smokers	0	202	19.2		1	
	1	72	18.9	0.90	0.87 (0.63–1.21)	0.42
	> 1	25	23.1	0.33	1.25 (0.74–2.09)	0.41
Cigarette per day by household smokers	0	202	19.2		1	
	1–20	82	19.1	0.89	0.90 (0.66–1.23)	0.51
	>20	41	22.6	0.56	1.01 (0.49–2.09)	0.98

a Rate of antibiotic use in each exposure variable. AOR: adjusted odds ratio; adjusted for children’s sex, age, body mass index (BMI) z score, history of breastfeeding, gestational age at birth, birthweight, maternal age, living region, overcrowding of living area, household income, child care attendance, and presence of siblings.

### Associations between ETS exposure and outcomes among children with a non-smoking mother

Associations between ETS exposure and health outcomes were further evaluated among children with non-smoking mothers only (N=1480, mother was neither smoking during pregnancy nor an active smoker). Household ETS exposure was significantly and independently associated with all-cause doctor consultation in the past 3 months (AOR=1.34; 95% CI: 1.06–1.70; p=0.021), even after exclusion of maternal smoking (Supplementary Tables 1 and 2).

## DISCUSSION

Young children with household ETS exposure were more likely to have all-cause medical service utilisation and recent respiratory symptoms compared with the non-exposed children. The risks were higher with increasing exposure levels. These findings suggest an increased healthcare burden associated with ETS exposure in young children, emphasizing the importance of identification, reduction, and prevention of ETS exposure in the paediatric population.

Our study found household ETS exposure was associated with both outpatient doctor consultation and hospitalisation in young children, while a previous local study demonstrated the association only in inpatient care but not in outpatient consultation^12^. In the previous study, infants with smoking mothers were excluded, doctor consultations and hospitalisation were dichotomized as either low or high use, and they coded infants as high users if the baby had higher utilisation than the median among the sample^12^. The difference in the methodology might explain the different study results. There are emerging studies showing the association of ETS with non-physical health outcomes, which may also be a reason for healthcare service utilization^26^.

The effect size of association between doctor visit and current household ETS exposure was relatively modest, while the effect size was stronger for postnatal maternal smoking. Postnatal maternal smoking was also associated with all-cause hospitalisation in the past 3 months. We did not detect significant independent associations between prenatal maternal smoking and all-cause medical service utilisation. However, the prevalence of maternal smoking was relatively low when compared with a previous study that showed a prevalence of 10.4%^27^. This study demonstrated that prenatal maternal smoking, of more than 15 cigarettes daily, is associated with a significant increase in the risk of the offspring being hospitalised for bronchiolitis^27^. Therefore, the absence of association in our study could be related to our small sample of prenatal maternal smoking. On the other hand, postnatal maternal smoking was significantly associated with doctor consultation in our cohort, which addressed the significant role of maternal smoking in children’s health. Maternal smoking behaviour could have a more direct impact on children’s health since mothers tend to smoke more at home, spend more time and have more contact with children, compared with other household members^11^.

Dose-dependent associations between ETS exposure and outcomes were observed in our study. Children living with more than one household smoker were more likely to have all-cause doctor consultation, and children living in a household with daily consumption of more than 20 cigarettes, were more likely to have respiratory symptoms compared with the non-exposed children. These findings have been rarely reported in the literature and this observation supports the causal relationship between ETS exposure and medical service utilisation. The toxicity and hazard effects of ETS on children have been well summarized in the literature^1^. ETS is not only responsible for respiratory illnesses but the hazard can be systemic^1,2^. Furthermore, a young child’s detoxification system is immature and thus less efficient in handling toxic components of ETS compared with those who are older^28,29^. Therefore, the harmful effects from ETS could be higher with higher level of exposure. A previous study reported similarly that children with high ETS exposure level were more likely to have had an overnight hospital stay, with the association strongest in asthmatic children^4^. They also found that children with higher exposure levels tend to have higher risk of healthcare utilisation^4^. Moreover, children in our cohort with higher ETS exposure were more likely to have recent respiratory symptoms. The association remained even after adjustment for recent respiratory symptoms of household members, which could be a significant confounding factor not considered in previous studies^22^. A previous study showed that ETS has little effect on the respiratory health of children aged 3–5 years but more effect on children aged <2 years and on children with asthma^22^. The relationship between ETS exposure and respiratory illness might interact with allergy^22^. Unfortunately, the children’s allergy history was not collected in our study. Other studies found ETS exposure increased the risk of chronic bronchitis, episodes of wheezing, and various respiratory symptoms such as cough, phlegm, and breathlessness, among children^15,30,31^.

There are multiple potential mechanisms by which ETS exposure causes respiratory illness. The lungs of young children are not fully developed until the age of 7 years^32^. The immature airway epithelium and respiratory tract infections create increased permeability of the epithelial layer of the respiratory tract, resulting in increased damage by ETS exposure^33^. Biomolecular studies also suggest that the development of the immune system will skew towards the T-helper 2 phenotype, increasing the susceptibility of developing allergy and respiratory symptoms with ETS exposure^28^.

ETS was found to be associated with higher risk of antibiotic use among children aged <5 years in several European studies^23,24^. The association might be related to the children’s family SES^23,24^. It can also be mediated with the higher risk of infectious diseases among ETS exposed children, while the mechanism by which ETS exposure increases susceptibility to infectious diseases is not fully understood^34^. However, no significant association was found between ETS exposure and antibiotic use in our study. The potential reason could be different medical practice on antibiotic use in different regions. Moreover, the information about recent antibiotic use and ETS exposure covered different time periods. It is possible that the practice of antibiotic use would change with time; more doctors now realize that antibiotics are not useful in fighting viral infections.

### Strengths and limitations

Strengths of our study are that it was representative of the Hong Kong territory, and that we provided new information in young children aged <18 months, less studied previously. Our analyses with exclusion of children with maternal smoking allowed the independent effect of household ETS exposure to be ascertained. There are a few limitations to address. Our analysis was based on our previous cross-sectional study, and the information was reported by the caregivers with the potential of recall bias. An objective measure of ETS exposure by cotinine level was not used. Potential under-reporting of smoking status could have diluted our power of identifying the health disparities between children with and without exposure. However, most of the participating caregivers of the children were the mothers of the children, and mothers’ proxy reports on the smoking status of the father were highly reliable^35^. Moreover, parental self-reported smoking status has been shown to be valid and reliable^35^. Finally, there might have been unmeasured or residual confounding, which cannot be excluded in our observational study.

## CONCLUSIONS

Our study demonstrated the associations of household ETS exposure with medical service utilisation and respiratory symptoms in young children. Policy makers, parents and healthcare providers have important roles in reducing children’s ETS exposure. We urge for more effective tobacco control policy and public health education. Healthcare providers should identify ETS exposure in children so that intervention and support can be provided.
