# Supplementary material for: Associations of household environmental tobacco smoke exposure with respiratory symptoms and utilisation of medical services in healthy young children in Hong Kong
Source: Tob Induc Dis. 2020 Jan 10;18:02. doi: 10.18332/tid/114461 (PMC6964497; doi:10.18332/tid/114461)
Supplement: Supplementary file 1 [file TID-18-02-s1.pdf]

Supplementary Table 1. Rates of medical care use associated with the ETS measures of the study participants in Hong Kong after exclusion of smoking mothers, 2013-2014 – Univariable analyses (N=1480)

ETS exposure measures		Have doctor consultation in the past 3 months			Have hospitalisation in the past 3 months			Presence of respiratory symptoms in the past 1 month			Antibiotic utilisation in the past 3 months		
		N	%	p value	N	%	p value	N	%	p value	N	%	p value
Presence of current household smoker (s)	No	620	59.0%		56	5.3%		341	32.5%		191	18.2%	
	Yes	281	65.3%	0.026	29	6.8%	0.32	156	36.3%	0.16	73	17.0%	0.60
Number of current household smoker (s)	0 household smoker	618	59.0%		55	5.2%		342	32.6%		190	18.1%	
	1 household smoker	239	64.9%	0.044	26	7.1%	0.19	128	34.8%	0.45	61	16.6%	0.50
	> 1 household smoker	44	68.8%	0.12	4	6.3%	0.73	27	42.2%	0.12	13	20.3%	0.66
Total number of cigarette consumption by current household smoker (s)	0 cigarette/ day	623	59.1%		56	5.3%		345	32.6%		193	18.3%	
	1-20 cigarettes/ day	251	64.5%	0.048	26	6.7%	0.32	134	34.4%	0.51	65	16.7%	0.48
	>20 cigarettes/ day	27	73.0%	0.094	3	8.1%	0.47	18	48.6%	0.045	6	16.2%	0.86

%: Rate of health outcomes in each exposure variable

© 2020 Dai S. and Chan K.C.
