# Supplementary material for: Associations of household environmental tobacco smoke exposure with respiratory symptoms and utilisation of medical services in healthy young children in Hong Kong
Source: Tob Induc Dis. 2020 Jan 10;18:02. doi: 10.18332/tid/114461 (PMC6964497; doi:10.18332/tid/114461)
Supplement: Supplementary file 2 [file TID-18-02-s2.pdf]

Supplementary Table 2. Associations between ETS exposure and health outcomes of the study participants in Hong Kong after exclusion of smoking mothers, 2013-2014 – multivariable analyses (N=1480)

ETS exposure measures		Have doctor consultation in the past 3 months		Have hospitalisation in the past 3 months		Presence of respiratory symptoms in the past 1 month		Antibiotic utilisation in the past 3 months	
		AOR (95% CI)	p value	AOR (95% CI)	p value	AOR (95% CI)	p value	AOR (95% CI)	p value
Presence of current household smoker (s)	No	1		1		1		1	
	Yes	1.34 (1.06, 1.70)	0.021	1.29 (0.79, 2.10)	0.32	1.19 (0.91, 1.55)	0.20	0.91 (0.67, 1.25)	0.91
Number of current household smoker (s)	0 household smoker	1		1		1		1	
	1 household smoker	1.30 (1.01, 1.68)	0.037	1.38 (0.83, 2.28)	0.21	1.09 (0.82, 1.44)	0.56	0.87 (0.63, 1.21)	0.87
	> 1 household smoker	1.57 (0.89, 2.77)	0.12	1.20 (0.40, 3.57)	0.75	1.68 (0.94, 3.02)	0.083	1.29 (0.66, 2.49)	0.46
Total number of cigarette consumption by current household smoker (s)	0 cigarette/ day	1		1		1		1	
	1-20 cigarettes/ day	1.27 (1.00, 1.64)	0.053	1.28 (0.77, 2.13)	0.34	1.09 (0.83, 1.44)	0.53	0.89 (0.65, 1.23)	0.49
	>20 cigarettes/ day	1.93 (0.91, 4.10)	0.094	1.72 (0.49, 6.03)	0.40	1.91 (0.92, 3.99)	0.086	0.81 (0.32, 2.01)	0.64

Adjusted factors: children’s sex, age, body mass index (BMI) z score, history of breastfeeding, gestational age at birth, birthweight, maternal age, living region, overcrowding of living area, household income, child care attendance and presence of siblings. Additional adjustment for season and households’ respiratory symptoms were made for association exploration between ETS and children’s respiratory symptoms

© 2020 Dai S. and Chan K.C.
